# A comparative analysis on characteristics and mortalities of four key transmission populations on antiretroviral therapy: a retrospective cohort study in Northwest China

**DOI:** 10.1186/s12879-022-07281-x

**Published:** 2022-03-28

**Authors:** Shuo Feng, Zirong Zhu, Pengju Yang, Juan Jin, Huihui Tuo, Ning Wang, Ruimin Bai, Yan Sun, Liumei Song, Xiu Zhang, Shengbang Wang, Qiqi Duan, Yingjian Huang, Yan Zheng, Songhua Xu

**Affiliations:** 1 Department of Dermatology, The Second Affiliated Hospital of Xi’an Jiaotong University, 157 Xiwu Road, Xi’an, Shaanxi Province China; 2 Department of Infectious Diseases, The Eighth Hospital of Xi’an Municipality, 2 Zhangbadong Road, Xi’an, Shaanxi Province China; 3 Institute of Medical Artificial Intelligence, The Second Affiliated Hospital of Xi’an Jiaotong University, 157 Xiwu Road, Xi’an, Shaanxi Province China

**Keywords:** HIV/AIDS, Antiretroviral therapy, Transmission category, Homosexuals, Cohort study

## Abstract

**Background:**

This study explored disparities in characteristics and mortalities among four major transmission groups on antiretroviral therapy in northwest China as well as the survival impact of each transmission route.

**Methods:**

We first examined disparities in demographics and clinical characteristics of the four transmission populations. Kaplan Meier analysis was subsequently conducted to compare survival rates among all groups. At last, Cox proportional hazards regression model was employed to analyze the survival impact of a transmission route among seven main categories of survival factors associated with all-cause mortalities.

**Results:**

Survival analysis showed significant differences in all-cause, AIDS- and non-AIDS-related deaths among four HIV populations (all *P* < 0.05). Using homosexuals as the reference, Cox proportional hazards model further revealed that the risk of all-cause death for blood and plasma donors was significantly higher than that of the reference (aHR: 5.21, 95%CI: 1.54–17.67); the risk of non-AIDS-related death for heterosexuals (aHR: 2.07, 95%CI: 1.01–4.20) and that for blood and plasma donors (aHR: 19.81, 95%CI: 5.62–69.89) were both significantly higher than that of the reference.

**Conclusions:**

Significant disparities were found in characteristics and mortalities among the four transmission groups where mortality disparities were mainly due to non-AIDS-related death. Suggestions are provided for each group to improve their survivorship.

**Supplementary Information:**

The online version contains supplementary material available at 10.1186/s12879-022-07281-x.

## Background

Antiretroviral Therapy (ART) can greatly reduce mortality and increase the life expectancy of people living with Human Immunodeficiency Virus (HIV) or Acquired Immunodeficiency Syndrome (AIDS) [1–3]. A nationwide study in China [4] found immediate ART for people living with HIV (PLWH) with CD4 positive lymphocyte counts larger than 500 cells/μL can reduce all-cause mortality by 63%. According to Joint United Nations Programme on HIV/AIDS (UNAIDS), approximately 38.0 million individuals globally were living with HIV in 2019, and 25.4 million PLWH (66.8%) were receiving ART [5]. It’s still a great challenge for many countries to reach the 95-95-95 target set by UNAIDS by 2025 [6].

Since “Four Frees and One Care policy” was implemented in mainland China from 2003, significant advance has been achieved in the prevention and treatment of HIV [7]. In 2016, China updated its national ART guideline which recommended ART for all PLWH regardless of their World Health Organization (WHO) HIV clinical stages or CD4 + T lymphocyte counts [8]. Before 2006, injecting drug users (IDUs) and former plasma donors (FPDs) were the vulnerable populations for HIV infection in China [9, 10]. In recent years, heterosexual contact has become the major route of transmission in China [11]. In the past few years, the domination of heterosexual transmission has shifted to that via homosexual contact in some economically developed areas of the nation, such as Shenzhen and Shanghai [12, 13].

PLWH are composed of heterogeneous groups with multiple sociodemographic characteristics. The political, economic and cultural differences in every region and the differences in characteristics of PLWH in every period both affect the disease outcomes. In the past, studies on HIV progression and mortality in China mainly concentrated on IDUs and FPDs, while few studies focused on homosexuals [14, 15]. Despite abundant studies taking place internationally for HIV transmission routes and the associated populations [16–20], the topic is sparsely visited for PLWH in China to guide the nation’s disease prevention, treatment and care. A study focused on PLWH in the Jiangsu Province of China from 2004 to 2010, China showed that men who have sex with men (MSM) progressed faster than heterosexuals and FPDs, but the all-cause mortality among MSM was lower [21]. A peer study focused on PLWH in the Zhejiang Province of China from 2006 to 2013 found that mortalities both related and unrelated to AIDS were lower for MSM than heterosexual individuals [22]. However, the above two studies did not consider ART in exploring the survival of PLWH. For PLWH receiving ART, the impact of the transmission route on mortalities is unknown. Among PLWH receiving ART, it has been reported that infection through homosexual contact was a protective factor for AIDS-related deaths, while infection through injecting drug use was a risk factor, both using the heterosexual group as a reference [23]. The risk of all-cause and non-AIDS-related mortality for each transmission population in China while receiving ART remains to be more thoroughly studied, whose findings can undoubtedly enable policymakers to make better informed decisions and take targeted measures to improve the quality of life and treatment outcomes for PLWH.

Xi’an, the capital of Shaanxi Province, is located in the northwest of China. The study used follow-up survey data from 5942 PLWH who received free ART from January 2010 to June 2019 at the Eighth Hospital of Xi’an Municipality, which is the only hospital designated in Xi’an for delivering free ART. Our study was designed to explore disparities in characteristics and mortalities among four major transmission groups on ART in northwest China as well as the survival impact of each transmission route. The end purpose aims at providing better guidance for the prevention and treatment of each of the four transmission populations.

## Methods

### Study design and participants

**Fig. 1:**
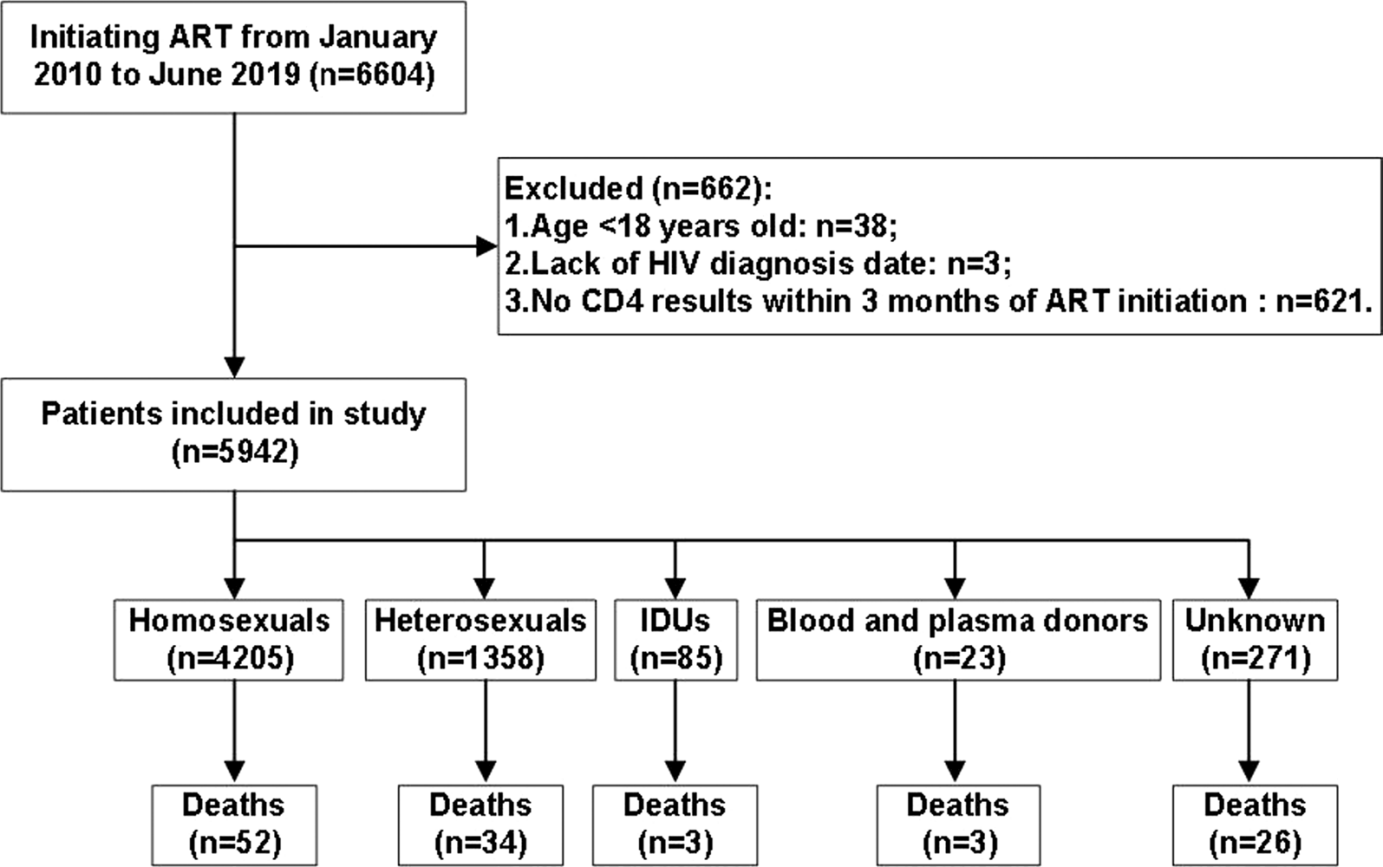
Flow chart of Inclusion criteria of the participants. *ART* antiretroviral therapy, *IDU* injecting drug user

This is a retrospective cohort study. 6604 PLWH received free ART in the Eighth Hospital of Xi’an Municipality from January 1, 2010, to June 30, 2019. The follow-up study started the data collection when a patient initiated his/ her ART until the study endpoint (June 30, 2019) or the date of death, loss of contact or withdrawal by the patient, whichever happened first. The inclusion criteria of the cohort were as follows: (1) the patient needs to be at least 18 years old; (2) having a clear HIV diagnosis date; (3) having a CD4 positive lymphocyte count test result within 3 months of ART initiation. Applying the above criteria, 5942 PLWH were included in this study, and were subsequently divided into five groups according to each person’s transmission route, including homosexual contact (4205), heterosexual contact (1358), drug injection (85), blood transfusion and plasma donor (23) and unknown routes (271) (Fig. 1). We carried out the study to explore disparities in characteristics and mortalities among four major transmission groups on ART as well as the survival impact of each transmission route.

### Data source and collection

Local healthcare professionals collected a range of information concerning the demographics and clinical characteristics of each patient throughout the study period, accompanied by the patient’s follow-up status, as well as cause and date of death, if applicable. The demographics information includes gender, date of birth, marital status, height, and weight; the clinical characteristics comprises date of diagnosis, date of ART initiation, self-reported transmission route, WHO clinical stage, CD4 positive T lymphocyte counts, comorbidities, as well as laboratory results regarding serum hepatitis B surface antigen (HbsAg) and hepatitis C virus (HCV) antibodies at ART initiation. The WHO clinical stage was classified based on the revised WHO guidelines for adults [24]. The list of comorbidities considered includes: (1) pulmonary tuberculosis, (2) extrapulmonary tuberculosis, (3) pneumocystis pneumonia, (4) recurrent severe bacterial pneumonia, (5) recurrent severe bacterial infection (other than pneumonia), (6) disseminated non-tuberculous mycobacterial infection, (7) disseminated mycoses, (8) persistent or intermittent fever (more than 1 month), (9) prolonged diarrhoea (more than 1 month), (10) thrush, (11) herpes zoster, 12) chronic herpes simplex, (13) Kaposi’s sarcoma, (14) oral hairy leukoplakia, (15) other skin lesions, (16) esophagal candidiasis, (17) extrapulmonary cryptococcosis, (18) cytomegalovirus infection, (19) central nervous system toxoplasmosis, (20) cerebral lymphoma, (21) B cell non-Hodgkin lymphoma, and (22) other opportunistic infections or tumours. A patient’s cause of death, if applicable, was codified according to the tenth edition of the International Classification of Diseases (ICD-10) [25] and determined based upon records maintained at the patient’s terminal care facility and supplemented by the Center for Chinese Disease Control and Prevention, family members or other contacts of the patient, if necessary. Such causes were subsequently aggregated into four main categories for the purpose of this study, including (1) AIDS-related deaths, such as AIDS-related tumours, opportunistic infections as well as other symptomatic diseases and syndromes, (2) non-AIDS-related deaths, such as non-AIDS-related tumours, respiratory diseases, cardiovascular diseases, cerebrovascular diseases, endocrine diseases, and digestive system diseases, (3) external deaths, such as drug overdose, drug toxicity, suicide, and accident, and (4) unknown cause of deaths.

### Statistical analysis

Descriptive and inferential statistical analyses were both conducted using the software SPSS Version 22.0 (SPSS Inc., Chicago, IL, USA). Continuous variables were reported as median [IQR (interquartile range)] and categorical variables were presented as number (percentage). Disparities in demographics and clinical characteristics among the four transmission groups were determined using the Kruskal–Wallis H test for the continuous variables and the Pearson Chi-square test for the categorical variables where *P*-values < 0.05 were considered statistically significant. The differences between any two groups were assessed using the Mann–Whitney U test for the continuous variables and the Pearson Chi-square test for the categorical variables. The Bonferroni correction was used in the multiple comparison test among the four transmission groups where the adjusted significance level was set to 0.008 [26, 27]. Mortality was calculated as person-years (py) of follow-up per thousand py with a 95% confidence interval (CI) for the specific causes of death among the four transmission populations. Kaplan Meier analysis and log-rank test were further applied to compare the survival rates among these groups. Lastly, Cox proportional hazards regression model was employed to analyze the survival impact of a transmission route among seven main categories of survival factors associated with all-cause mortalities. All hypothesis tests were two-sided where *P*-values < 0.05 was considered statistically significant.

## Results

### Trend in dominant HIV transmission route

**Fig. 2:**
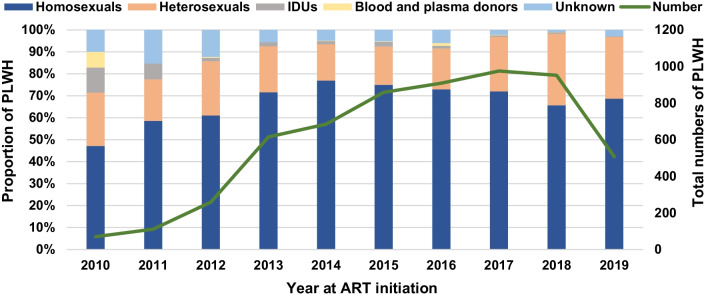
The proportion of PLWH stratified by transmission category (left) from January 2010 to June 2019 along with the number of PLWH initiating ART in each year (right). Note that for 2019, the number of PLWH drops because only half year’s data is available. *PLWH* people living with HIV, *ART* antiretroviral therapy, *IDU* injecting drug user

Among the 5942 PLWH included in the study, 70.8% of them acquired HIV via homosexual contact. During the study period, homosexual infection cases increased rapidly in numbers. Only 33 cases in homosexuals were reported in 2010, while the number escalated to 702 in 2017 and declined modestly to 625 in 2018. In terms of proportions, the proportion of heterosexual infection cases rose annually since 2014, accompanied by the decline of the proportion of homosexual infection cases in the meanwhile. In 2010, the proportion of IDUs was 11.4%, which fell rapidly to 1.5% in 2012 and remained at the level afterwards. Through the study period, only 23 cases of blood and plasma donor infection were witnessed and no cases of mother-to-infant vertical transmission were encountered. The dynamics in the proportion of PLWH stratified by transmission category are illustrated in Fig. 2.

### Baseline characteristics

**Fig. 3:**
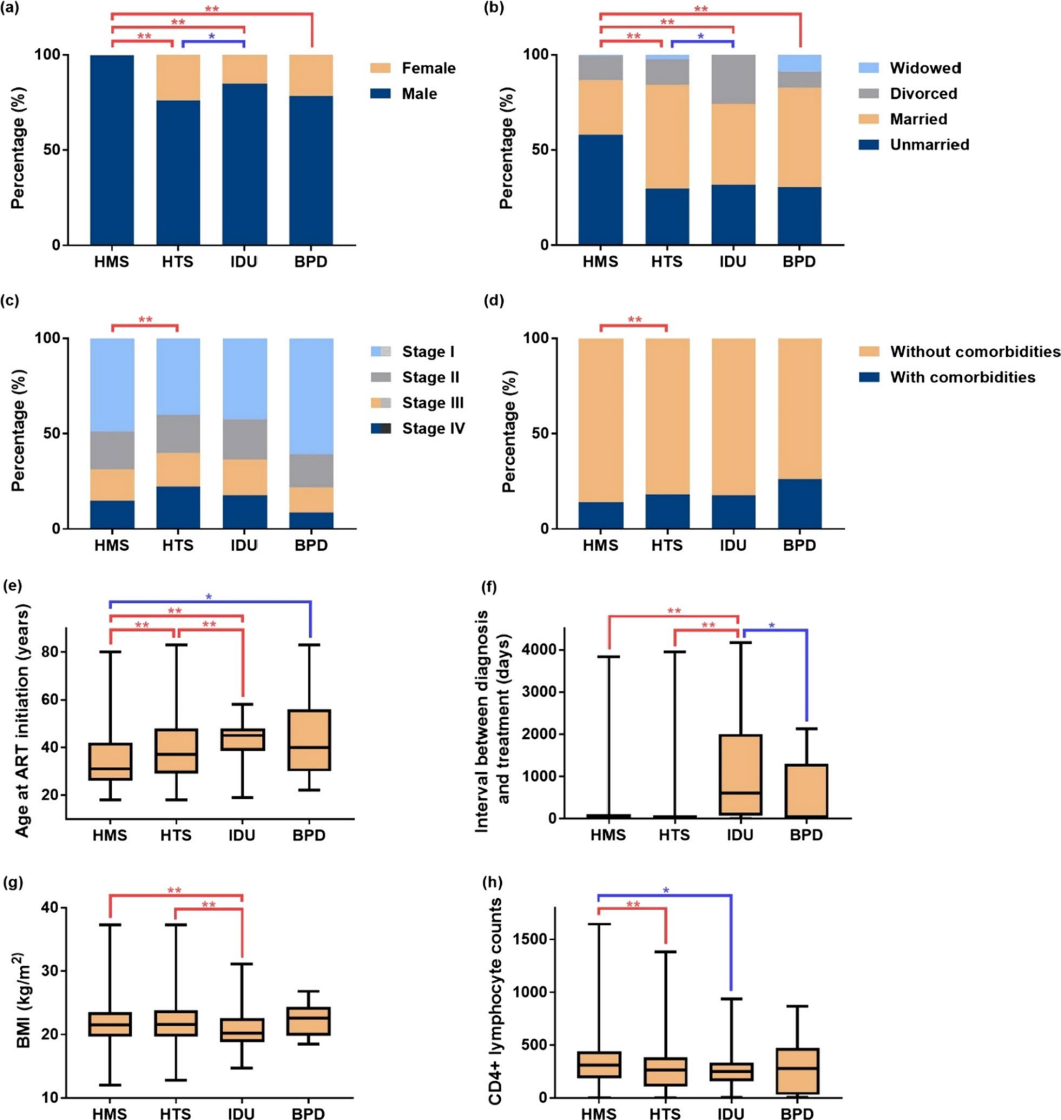
Comparing of baseline characteristics among four transmission categories, including (**a**) gender, (**b**) marital status, (**c**) WHO clinical stage, (**d**) comorbidity, (**e**) age at ART initiation, (**f**) interval between HIV diagnosis and ART initiation, (**g**) BMI, and (**h**) CD4 + lymphocyte counts. (Statistically significant pairwise comparison results are indicated using ∗ and highlighted in blue if 0.001 < *P* < 0.008 or indicated using ∗ ∗ and highlighted in red if *P* < 0.001. *HMS* homosexuals, *HTS* heterosexuals, *IDU* injecting drug user, *BPD* blood and plasma donors, *ART* antiretroviral therapy, *BMI* body mass index)

As shown in Table 1, among all PLWH, the male cases accounted for 93.2% while the female cases only 6.8%, with a male-to-female gender ratio of 13.5 (5532/ 410). Among male patients, homosexual contact was the main route of transmission (57.9%), whereas, among female patients, heterosexual contact was the main transmission route (54.7%). Most of the cases were unmarried, accounting for 49.1%. Significant statistical differences existed in marital status among the four population groups (*P* < 0.05), in that homosexuals were predominantly unmarried (57.9%), while the majority of heterosexuals and IDUs were married (54.7% and 42.4%, respectively). In terms of age, most of PLWH were between 18 and 39 years (65.6%), with an average age of 33 years (IQR 27–44 years). As shown in Fig. 3, homosexuals started receiving ART at the youngest age among all groups; IDUs started ART at an older age compared with heterosexuals, who also experienced the longest delay in receiving ART after their HIV diagnosis compared with the rest of groups; the IDU group has much lower body mass index (BMI) than those of the homosexual and heterosexual groups; homosexuals have much higher CD4 positive T lymphocyte counts at their ART initiation than heterosexuals and IDUs. All above findings were statistically significant with P < 0.05 after applying the Bonferroni correction.

**Table 1:** Baseline characteristics of PLWH stratified by transmission category

	All	Homosexuals	Heterosexuals	IDUs	Blood and plasma donors	Unknown^b^	*P-*value^c^
Number	5942	4205	1358	85	23	271	
Gender* [number (%)]							
Male	5535 (93.2)	4198 (99.8)	1034 (76.1)	72 (84.7)	18 (78.3)	213 (78.6)	** < 0.001**
Female	407 (6.8)	7 (0.2)	324 (23.9)	13 (15.3)	5 (21.7)	58 (21.4)	
Marital status^a^* [number (%)]							
Unmarried	2917 (49.1)	2433 (57.9)	402 (29.6)	27 (31.8)	7 (30.4)	48 (17.7)	** < 0.001**
Married	2173 (36.6)	1205 (28.7)	743 (54.7)	36 (42.4)	12 (52.2)	177 (65.3)	
Divorced	775 (13.0)	538 (12.8)	180 (13.3)	22 (25.9)	2 (8.7)	33 (12.2)	
Widowed	74 (1.20)	28 (0.7)	33 (2.4)	–	2 (8.7)	11 (4.1)	
Unknown^b^	3 (0.1)	1 (0.0)	–	–	–	2 (0.7)	
Age at ART initiation*							
Years, median (IQR)	33 (27–44)	32 (26–42)	37 (29–48)	45 (39–48)	40 (30–56)	45 (33–57)	** < 0.001**
Interval between diagnosis moment and ART initiation*							
Days, median (IQR)	33 (18–105)	33 (18–105)	33 (19–85)	603 (74–2005)	36 (15–1301)	33 (15–85)	** < 0.001**
BMI^a^* (kg/ m^2^)							
Median (IQR)	21.5 (19.6–23.6)	21.5 (19.7–23.5)	21.6 (19.7–23.8)	20.2 (18.8–22.6)	22.6 (19.8–24.4)	21.1 (19.2–23.2)	**0.002**
CD4 + lymphocyte counts^a^* (/μL)							
Median (IQR)	295 (160–425)	311 (186–442)	265 (108–385)	250 (160–334)	280 (31–472)	196 (56–315)	** < 0.001**
WHO clinical stage^a^* [number (%)]							
Stage I	2749 (46.3)	2056 (48.9)	544 (40.1)	36 (42.4)	14 (60.9)	99 (36.5)	** < 0.001**
Stage II	1175 (19.8)	836 (19.9)	272 (20.0)	18 (21.2)	4 (17.4)	45 (16.6)	
Stage III	991 (16.7)	690 (16.4)	239 (17.6)	16 (18.8)	3 (13.0)	43 (15.9)	
Stage IV	1027 (17.3)	623 (14.8)	303 (22.3)	15 (17.6)	2 (8.7)	84 (31.0)	
Comorbidities^a^* [number (%)]							
With comorbidities	972 (16.4)	592 (14.1)	245 (18.0)	15 (17.6)	6 (26.1)	114 (42.1)	**0.002**
Without comorbidities	4970 (83.6)	3613 (85.9)	1113 (82.0)	70 (82.4)	17 (73.9)	157 (57.9)	
HbsAg^a^ [number (%)]							
Positive	327 (5.5)	222 (5.3)	86 (6.3)	3 (3.5)	3 (13.0)	13 (4.8)	0.136
Negative	5019 (84.5)	3562 (84.7)	1138 (83.8)	69 (81.2)	17 (73.9)	233 (86.0)	
Unknown^b^	596 (10.0)	421 (10.0)	134 (9.9)	13 (15.3)	3 (13.0)	25 (9.2)	
Anti-HCV antibodies^a^ [number (%)]							
Positive	168 (2.8)	115 (2.7)	44 (3.2)	3 (3.5)	1 (4.3)	5 (1.8)	0.475
Negative	5149 (86.7)	3649 (86.8)	1175 (86.5)	70 (82.4)	19 (82.6)	236 (87.1)	
Unknown^b^	625 (10.5)	441 (10.5)	139 (10.2)	12 (14.1)	3 (13.0)	30 (11.1)	

*PLWH* people living with HIV, *IDU* injecting drug user, *IQR* interquartile range, *ART* antiretroviral therapy, *BMI* body mass index, *HbsAg* hepatitis B surface antigen and HCV, hepatitis C virus

^a^Marital status, BMI, WHO clinical stage, CD4 + T lymphocyte counts, comorbidities and testing of HbsAg and anti-HCV antibodies were determined based on the date of ART initiation

^b^Patients with missing data were included in the unknown group

^c^*P*-values are calculated for comparisons among homosexuals, heterosexuals, IDUs and blood and plasma donors. Characteristics with statistically significant differences among the four transmission groups are selected for further analysis, which are indicated using a * in the superscript

### Mortality and causes of death

We followed up 5942 PLWH who represented 17770py. During the follow-up period, the number of deaths was 118, including 69 AIDS-related deaths, 43 non-AIDS-related deaths, 5 suicidal or accidental deaths, and 1 death with unknown causes. There were more AIDS-related deaths than non- AIDS-related deaths for homosexuals, heterosexuals, and the whole study population. As shown in Additional file 1: Fig. S1, between 2010 and 2019, all causes of mortality decreased from 122.1 per 1000 py (2010) to 4.0 per 1000 py (2019) among PLWH. The AIDS-related mortality was 91.6 per 1000 py (2010) among PLWH decreased to 2.4 per 1000 py (2019), and the non-AIDS-related mortality was 30.5 per 1000 py (2010) decreased to 1.6 per 1000 py (2019). According to results shown in Table 2, the crude all-cause mortality rate of the homosexuals (4.1/1000 py) was 0.4 times that of the heterosexuals (9.3/1000 py); while the weighted average of crude all-cause mortality rate for homosexuals (2.9/1000 py; 95% CI: 2.0–3.8/1000 py) is 1.4 times that of heterosexuals (2.1/1000 py; 95%CI: 1.3–2.9/1000 py), showing different death risks for the two main population groups and their disproportional death counts. Among our follow-up data, only three deaths occurred in the IDU group, all of which were related to AIDS; in contrast, the blood and plasma donor group also had only three deaths, all of which were non- AIDS-related.

**Table 2:** Mortality rates for specific causes of death stratified by transmission category

Causes of death	n	py	All-cause		AIDS-related		Non-AIDS-related		External		Unknown	
			Deaths	WA (95%CI)	Deaths	WA (95%CI)	Deaths	WA (95%CI)	Deaths	WA (95%CI)	Deaths	WA (95%CI)
Total	5942	17,770	118	6.6 (5.2–8.0)	69	3.9 (2.8–5.0)	43	2.4 (1.6–3.3)	5	0.3 (0.0–0.6)	1	0.1 (0.0–0.2)
Homosexuals	4205	12,776	52	2.9 (2.0–3.8)	31	1.7 (1.0–2.4)	17	0.9 (0.4–1.5)	3	0.2 (0.0–0.4)	1	0.1 (0.0–0.2)
Heterosexuals	1358	3669	34	2.1 (1.3–2.9)	18	1.1 (0.5–1.7)	14	0.9 (0.4–1.4)	2	0.1 (0.0–0.3)	–	–
IDUs	85	246	3	0.2 (0.0–0.4)	3	0.2 (0.0–0.4)	–	–	–	–	–	–
Blood and plasma donors	23	83	3	0.1 (0.0–0.3)	–	–	3	0.1 (0.0–0.3)	–	–	–	–
Unknown	271	996	26	1.2 (0.6–1.8)	17	0.8 (0.3–1.3)	9	0.4 (0.1–0.8)	–	–	–	–

*py* person-years, *WA* weighted average of crude mortality rate, *CI* confidence intervals, *IDU* injecting drug user, *HbsAg* hepatitis B surface antigen and HCV, hepatitis C virus

WA = (deaths/ py) × (n/5942); deaths/ py = mortality rate

### Survival analysis

**Fig. 4:**
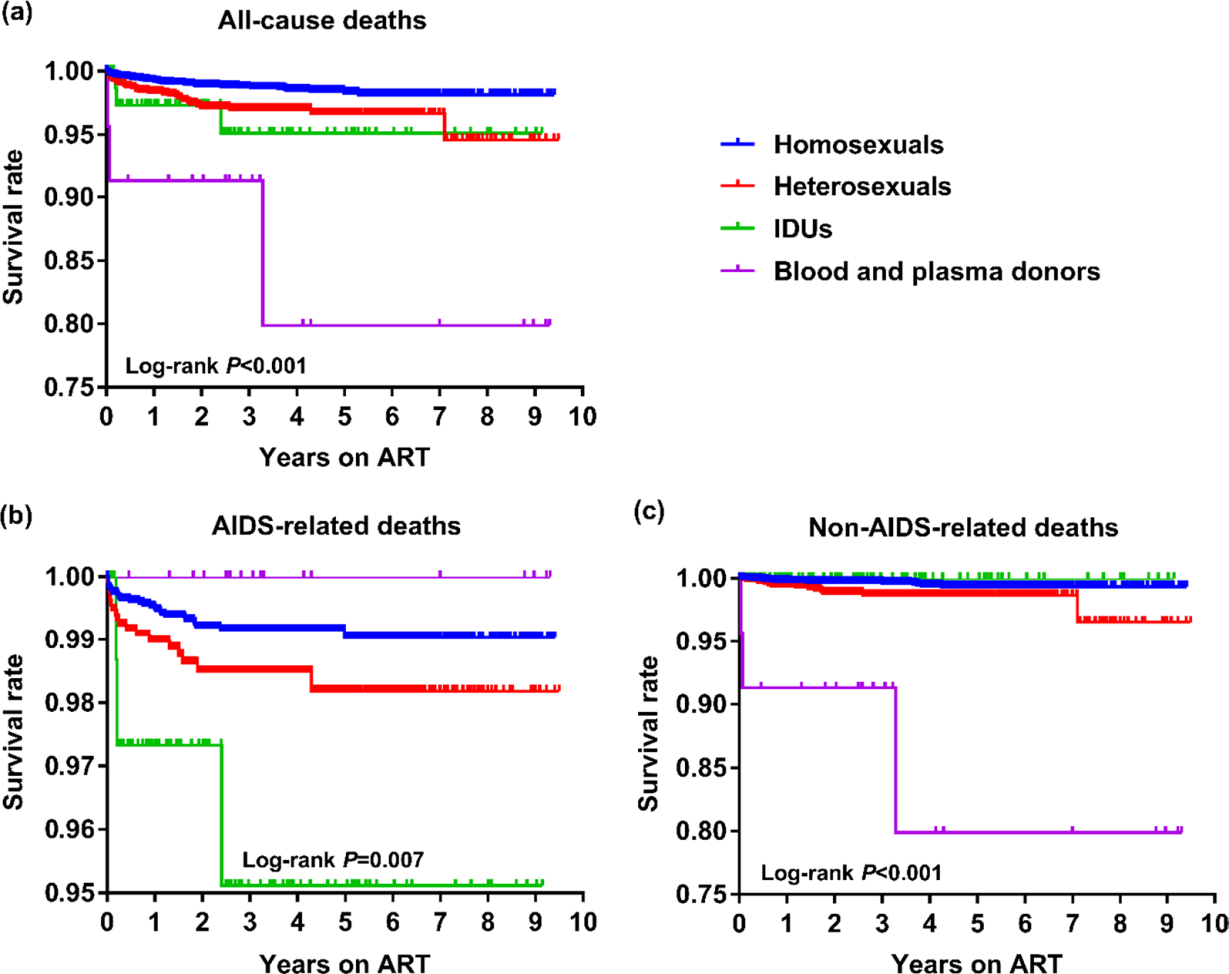
Kaplan–Meier survival curves for the four transmission populations for all-cause mortality (**a**), AIDS-related mortality (**b**), non-AIDS-related mortality stratified by HIV transmission category (**c**). *ART* antiretroviral therapy, *IDU* injecting drug user

Kaplan Meier survival analysis and log-rank test showed significant differences in all-cause, AIDS- and non-AIDS-related deaths among four HIV populations (all *P* < 0.05), among which the homosexuals had the lowest all-cause mortality, followed by heterosexuals with a higher rate, then the other two populations (all *P* < 0.05) (Fig. 4). Using homosexuals as the reference, both heterosexuals and IDUs had significantly higher AIDS-related mortalities while both heterosexuals and blood and plasma donors had significantly higher non-AIDS-related mortalities (all *P* < 0.05). In particular, among the four transmission groups, the blood and plasma donor group had the highest non-AIDS-related mortality (all *P* < 0.05).

### Survival impact of each transmission route

As shown in Table 3, through univariate analysis, seven main categories of survival factors associated with all-cause mortalities were identified, including gender, age at ART initiation, marital status, transmission category, BMI, CD4 positive lymphocyte counts and types of comorbidities (*P* < 0.05). Utilizing multivariate analysis, we further found that for PLWH receiving ART, transmission category was recognized as one of the factors affecting all-cause and non-AIDS-related deaths but not AIDS-related deaths. Using homosexuals as the reference, Cox proportional hazards model further revealed that the risk of all-cause death for blood and plasma donors was significantly higher than that of the reference (aHR: 5.21, 95%CI: 1.54–17.67); the risk of non-AIDS-related death for heterosexuals (aHR: 2.07, 95%CI: 1.01–4.20) and that for blood and plasma donors (aHR: 19.81, 95%CI: 5.62–69.89) were both significantly higher than that of the reference (Table 3).

**Table 3:** Cox proportional hazards regression models for mortality and associated risk factors

	All-cause deaths		AIDS-related deaths		Non-AIDS-related deaths	
	Univariate analysis	Multivariate analysis	Univariate analysis	Multivariate analysis	Univariate analysis	Multivariate analysis
Gender						
Male	1.00	–	–	–	1.00	–
Female	1.93 (1.10–3.37)	–	–	–	3.83 (1.83–7.98)	–
Age at ART initiation						
18–29	1.00	1.00	1.00	–	1.00	1.00
30–39	2.89 (1.38–6.08)	2.02 (0.95–4.30)	2.89 (1.19–7.03)	–	1.88 (0.31–11.23)	1.43 (0.24–8.61)
≥ 40	9.20 (4.78–17.72)	**5.62 (2.85–11.10)**	7.13 (3.22–15.78)	–	20.55 (4.96–85.22)	**14.10 (3.37–59.12)**
Marital status						
Unmarried	1.00	–	1.00	–	1.00	–
Married	3.48 (2.20–5.51)	–	2.63 (1.49–4.62)	–	8.96 (3.14–25.56)	–
Divorced	3.04 (1.69–5.48)	–	2.74 (1.34–5.60)	–	6.67 (1.95–22.80)	–
Widowed	10.46 (4.29–25.52)	–	4.89 (1.13–21.07)	–	43.28 (10.81–173.30)	–
Transmission category						
Homosexuals	1.00	1.00	1.00	–	1.00	1.00
Heterosexuals	2.15 (1.40–3.31)	1.49 (0.97–2.31)	1.89 (1.06–3.37)	–	2.75 (1.36–5.59)	**2.07 (1.01–4.20)**
IDUs	3.01 (0.94–9.64)	1.25 (0.39–4.06)	5.07 (1.55–16.59)	–	–	–
Blood and plasma donors	9.94 (3.01–31.92)	**5.21 (1.54–17.67)**	–	–	29.67 (8.61–102.29)	**19.81 (5.62–69.89)**
BMI (kg/ m^2^)						
< 18.5	1.00	1.00	1.00	–	–	–
18.5–24.9	0.40 (0.27–0.60)	**0.62 (0.40–0.96)**	0.46 (0.27–0.79)	–	–	–
25–19.9	0.20 (0.08–0.46)	**0.39 (0.16–0.97)**	0.12 (0.03–0.51)	–	–	–
≥ 30	0.66 (0.16–2.76)	1.56 (0.36–6.76)	0.60 (0.08–4.49)	–	–	–
CD4 + lymphocyte counts (/μl)						
< 50	1.00	1.00	1.00	1.00	1.00	–
50–99	0.71 (0.42–1.21)	0.87 (0.51–1.50)	0.63 (0.33–1.22)	0.78 (0.40–1.52)	0.92 (0.34–2.49)	–
100–199	0.50 (0.31–0.81)	0.94 (0.56–1.57)	0.38 (0.20–0.71)	0.67 (0.350–1.30)	0.95 (0.42–2.15)	–
200–349	0.13 (0.08–0.23)	**0.32 (0.17–0.59)**	0.10 (0.05–0.20)	**0.21 (0.09–0.46)**	0.21 (0.08–0.53)	–
350–499	0.04 (0.02–0.12)	**0.15 (0.05–0.44)**	–	–	0.18 (0.06–0.57)	–
≥ 500	0.06 (0.02–0.17)	**0.25 (0.09–0.75)**	–	–	0.19 (0.05–0.70)	–
Types of comorbidities						
0	1.00	1.00	1.00	1.00	1.00	1.00
1	6.10 (3.95–9.43)	**2.66 (1.64–4.30)**	9.25 (5.26–16.24)	**3.92 (2.14–7.17)**	3.99 (1.91–8.34)	**2.70 (1.27–5.74)**
2	10.53 (6.26–17.69)	**3.61 (2.03–6.40)**	13.74 (6.92–27.31)	**4.61 (2.20–9.65)**	7.58 (3.19–17.99)	**5.27 (2.19–12.66)**
≥ 3	13.41 (7.34–24.51)	**3.67 (1.86–7.26)**	14.70 (6.26–34.480)	**4.46 (1.81–10.98)**	9.91 (3.69–26.66)	**6.75 (2.42–18.87)**

*IDU* injecting drug user, *ART* antiretroviral therapy, *BMI* body mass index. Boldface indicates statistical significance

## Discussion

As analyzed above, during the follow-up period, homosexual contact has become the primary transmission route in Xi’an. It is anticipated that the real proportion of homosexuals among the study population would likely be even higher because of distorted self-report due to cultural stigma attached to the group [28]. In this study, MSM made up 99.8% (4198/ 4205) of homosexuals. We also found that among all MSM covered in the study population, 28.7% (1205/ 4198) were married to female wives. Such marriages were likely established due to cultural pressures, considerations for childbirth and bisexuality, thus incurring a high transmission risk among the couples. It has been previously reported that bisexual behaviour has become a major risk factor for homosexuals to spread HIV to the general population [29]. Homosexuals started receiving ART at the youngest age among all groups, which may be related to their early infection or diagnosis dates, showing that the group collectively has better awareness and knowledge about HIV/AIDS and its testing than other peer groups. As confirmed by a study in Shanghai, China, MSM had a higher level of education than other patients [12]. In our study, homosexuals had fewer clinical events and higher CD4 + T lymphocyte counts at ART initiation. Su et al. concluded that MSM who had high CD4 + T lymphocyte counts at ART initiation attain better treatment effects than other patients [23]. Early ART initiation can reduce sexual transmission of HIV and the incidence of clinical events, leading to both personal and public health benefits [30]. All factors discussed in the above can cast a positive impact on the prevention and control of HIV for homosexuals. Given the high HIV prevalence in MSM and the relatively fast transmission rate among the group due to large-scale MSM networks, efforts are particularly needed for preventing the disease transmission among MSM [31, 32]. One immediate strategy is to strengthen sex education through MSM networks by passing on basic knowledge necessary to prevent and manage HIV/AIDS as well as emphasizing the importance of early ART initiation.

Despite effective ART, PLWH still experience substantial mortality with increased prevalence of age-related, non-AIDS diseases [33]. Findings from past studies suggested infection by heterosexual contact was one of the main risk factors related to non-AIDS-associated mortality compared to infection by homosexual contact, which further supported our results [22]. For heterosexuals, the risk of non-AIDS-related deaths greatly exceeded that of homosexuals, likely owing to their lower baseline CD4 + T lymphocytes, more comorbidities and higher WHO clinical stage at ART initiation. Previous studies have shown heterosexuals achieved satisfactory immunological status more slowly than homosexuals [34]. Additionally, the higher risk of all-cause and non-AIDS-related death in the blood and plasma donor group with respect to the reference group may be attributed to their older age at ART initiation, higher comorbidity prevalence, and higher rates of HBV and HCV infections [35]. In the unadjusted analysis, heterosexuals and IDUs had a higher hazard of AIDS-related death than homosexuals. It is noteworthy that after adjusting for age, marital status, BMI, CD4 positive lymphocyte counts, and comorbidities, we did not observe any significant AIDS-related mortality differences among these four groups, the finding of which suggests that group-level mortality differences were mainly due to non-AIDS-related causes.

Except for the IDU group, about 80% of PLWH in the other three groups started ART within 6 months after diagnosis, whilst 62.4% of IDUs had more than 1 year’s delay in receiving ART after their diagnoses. The scope of ART has been gradually expanding in China since the changes in relevant policies occurred after 2003 [36]. Transmission through drug injection in China took place primarily before the launch of this study when the free ART program had not been administrated nationally in China, which likely caused a delay in ART for IDUs, leading to a higher death rate for the group [9, 36]. A large cohort study in Barcelona, Spain showed that heterosexuals had a higher risk of non-AIDS-related death than MSM, with which the results of this study agree [16]. Another cohort study in southwest China reported that poor compliance for IDUs led to increased mortality [37]. Therefore, our prevention strategies for IDUs should be emphasizing on shortening the interval between diagnosis and treatment as well as enhancing the compliance of IDUs during ART.

This study has several limitations. First, this is a retrospective cohort study using data collected from a national ART database, which inevitably carries missing and erroneous records. Second, information bias may likely exist due to distorted self-report of HIV transmission category due to cultural stigma. Third, low HIV prevalence among IDUs and FPDs in Xi’an led to a small sample size for the IDU and blood transmission groups, preventing effectively analyzing characteristics of the population. Fourth, the study is limited to one geographical area or municipality, which may have contributed to the findings. Lastly, policy changes regarding ART administration in China during our study period may have influenced the delays between HIV diagnosis and ART initiation.

## Conclusions

Significant disparities were found in characteristics and mortalities among the four transmission groups; for the latter, the disparities were mainly due to non-AIDS-related death. The HIV epidemic spreads from the high-risk population to the general population. Failure to control the spread of HIV will inevitably pose a huge threat to children, spouses and sexual partners. Suggestions are provided for each group to improve their survivorship as follows: for homosexuals, the proposed prevention strategies should concentrate on reducing HIV transmission; for heterosexuals, future management of the PLWH should focus on early diagnosis, timely and effective ART, and treatment of comorbidities; for IDUs, it is especially critical to encourage them to initiate ART timely after diagnosis; and for blood and plasma donors, the treatment of hepatitis virus infection and comorbidities needs to be strengthened to reduce the groups’ non-AIDS-related mortalities. China’s Free ART program provides reliable and well-funded resources to combat HIV in the nation. In particular, the program can help curb HIV prevalence in the population, which in turn reduces the incidence of HIV infections and propels attaining the 95-95-95 target sooner [6].

## Supplementary Information

**Additional file 1: Supplementary Figure S1.** Mortality rate among PLWH receiving ART in 2010–2019.

## Data Availability

The data that support the findings of this study are available from AIDS Prevention and Control Information System. However, restrictions apply to the availability of these data, which were used under license for the current study. So these data are not publicly available. For data acquisition, please contact the corresponding author.

## Abbreviations

ART: Antiretroviral therapy
HIV: Human immunodeficiency virus
AIDS: Acquired immunodeficiency syndrome
PLWH: People living with HIV
UNAIDS: Joint United Nations Programme on HIV/AIDS
WHO: World Health Organization
IDU: Injecting drug user
FPD: Former plasma donor
MSM: Men who have sex with men
HbsAg: Hepatitis B surface antigen
HCV: Hepatitis C virus
IQR: Interquartile range
py: Person-years
CI: Confidence interval
BMI: Body mass index
aHR: Adjusted hazard ratio
