# Supplementary material for: A comparative analysis on characteristics and mortalities of four key transmission populations on antiretroviral therapy: a retrospective cohort study in Northwest China
Source: BMC Infect Dis. 2022 Mar 28;22:299. doi: 10.1186/s12879-022-07281-x (PMC8962555; doi:10.1186/s12879-022-07281-x)
Supplement: Supplementary file 1 — Additional file 1: Supplementary Figure S1. Mortality rate among PLWH receiving ART in 2010–2019. [file 12879_2022_7281_MOESM1_ESM.docx]

**Supplementary Figure S1.** Mortality rate among PLWH receiving ART in 2010- 2019. (Abbreviation: PLWH, people living with HIV; ART, antiretroviral therapy.)
